# Assimilation of MODIS Snow Cover Fraction Observations into the NASA Catchment Land Surface Model

**DOI:** 10.3390/rs10020316

**Published:** 2018-02-19

**Authors:** Ally M. Toure, Rolf H. Reichle, Barton A. Forman, Augusto Getirana, Gabrielle J. M. De Lannoy

**Affiliations:** 1 Wilfrid Laurier University, 75 University Ave W, Waterloo, ON N2L 3C5, Canada; 2 Global Modeling and Assimilation Office, Code 610.1, NASA Goddard Space Flight Center, Greenbelt, MD, USA; rolf.h.reichle@nasa.gov; 3 Department of Civil and Environmental Engineering, University of Maryland, College Park, MD, USA; baforman@umd.edu; 4 Earth System Science Interdisciplinary Center, College Park, MD, USA; augusto.getirana@nasa.gov; 5 Hydrologic Sciences Laboratory, Code 617, NASA Goddard Space Flight Center, Greenbelt, MD, USA; 6 KU Leuven, Department of Earth and Environmental Sciences, Heverlee, Belgium; gabrielle.delannoy@kuleuven.be

**Keywords:** snow, land surface model, snow cover fraction, snow depth, snow water equivalent, data assimilation

## Abstract

The NASA Catchment land surface model (CLSM) is the land model component used for the Modern-Era Retrospective Analysis for Research and Applications (MERRA). Here, the CLSM versions of MERRA and MERRA-Land are evaluated using snow cover fraction (SCF) observations from the Moderate Resolution Imaging Spectroradiometer (MODIS). Moreover, a computationally-efficient empirical scheme is designed to improve CLSM estimates of SCF, snow depth, and snow water equivalent (SWE) through the assimilation of MODIS SCF observations. Results show that data assimilation (DA) improved SCF estimates compared to the open-loop model without assimilation (OL), especially in areas with ephemeral snow cover and mountainous regions. A comparison of the SCF estimates from DA against snow cover estimates from the NOAA Interactive Multisensor Snow and Ice Mapping System showed an improvement in the probability of detection of up to 28% and a reduction in false alarms by up to 6% (relative to OL). A comparison of the model snow depth estimates against Canadian Meteorological Centre analyses showed that DA successfully improved the model seasonal bias from −0.017 m for OL to −0.007 m for DA, although there was no significant change in root-mean-square differences (RMSD) (0.095 m for OL, 0.093 m for DA). The time-average of the spatial correlation coefficient also improved from 0.61 for OL to 0.63 for DA. A comparison against in situ SWE measurements also showed improvements from assimilation. The correlation increased from 0.44 for OL to 0.49 for DA, the bias improved from −0.111 m for OL to −0.100 m for DA, and the RMSD decreased from 0.186 m for OL to 0.180 m for DA.

## 1. Introduction

Seasonal snow cover has a significant impact on global climatological and hydrological processes [1]. For example, seasonal snow cover extent (SCE) exerts control over climate variability and change because of the physical properties of the snow cover, such as high albedo, high thermal infrared emissivity, low thermal conductivity, and latent heat sink [2]. In addition, due to the positive temperature-snow albedo feedback, snow acts to amplify perturbations in the global atmospheric circulation [3–5]. In the Northern Hemisphere, SCE ranges from an average maximum in January of 45.2 × 10^6^ km^2^ to an average minimum of 1.9 × 10^6^ km^2^ in August [6], with a generally decreasing trend observed in spring [7–9].

Snow acts as a frozen storage term in the water budget and is important for hydroelectric power, fresh water supply, irrigation, streamflow control, and flood preparedness. In mountainous regions of the Western United States (US) as much as 80% of the total runoff originates from snowmelt [10,11]. Predicting SCE, snow water equivalent (SWE), and the onset of melting is essential for hydrological and water resources management in many regions. Snow cover fraction (SCF), the fraction of unit land area covered by snow [12–14], is an important parameter that impacts the surface energy exchange, and, in the case of rain-on-snow events, determines how much liquid precipitation falls on snow-covered versus bare ground.

Numerous satellite-based sensors are used to detect seasonal snow cover, such as the Scanning Multichannel Microwave Radiometer (SMMR) [15], the Special Sensor Microwave/Imager (SSM/I) [16], the Landsat Thematic Mapper (TM) [17,18], the Advanced Very High Resolution Radiometer (AVHRR) [19], and the Moderate Resolution Imaging Spectroradiometer (MODIS) [20]. Satellite observations of snow cover extent are reasonably accurate [21–32] but are often discontinuous in space and time due to cloud cover, sensor type, or swath width limitations. Model predictions are continuous in time and space, but are limited by errors due to imperfect parameterization of complex natural processes and errors in forcing inputs and boundary conditions.

To make the most of two disparate information sources (i.e., observations and models), data assimilation techniques can be used to merge observations with model predictions for improved spatial and temporal coverage, consistency, resolution, and accuracy [33,34]. A large number of studies have been conducted that assimilate snow cover observations into Land Surface Models (LSMs) with different techniques and various degrees of success [35–46]. For example, Rodell and Houser [35] used a simple rule-based approach (a.k.a. direct-insertion) to assimilate MODIS SCF observations into the Mosaic land surface model [47]. A nominal amount of snow within a grid cell was added (or removed) from the model when the model and observation disagreed. Their study showed marginal improvements in SWE in low-altitude regions and in areas with ephemeral snow; however, assimilation often degraded model performance in areas with complex terrain at high altitude. The frequent cycle of the addition and subsequent melting of snow in these areas led to a large distortion in the modeled water and energy balance. Generally, the direct-insertion technique [35,36] is simple to implement and computationally efficient. However, the approach uses a nominal, fixed amount of SWE as increment and does not take into account the uncertainties in the observations or the model.

Alternatively, the Ensemble Kalman Filter (EnKF, [48]) uses an ensemble of model states to represent the uncertainties in the model estimate. The technique efficiently handles nonlinear models [49,50]. In [37], for example, an EnKF was used in conjunction with a rule-based scheme to assimilate Advanced Microwave Scanning Radiometer-EOS (AMSR-E) SWE and MODIS SCF observations into the Noah LSM [51] over a small domain (75 × 100 km^2^) in Northern Colorado. While the assimilation results showed some improvement in RMSE and correlation, the assimilation had marginal impact in areas of deep and complete snow cover. Furthermore, in [38] an EnKF assimilation scheme was compared to the rule-based scheme of [35] using MODIS SCF observations and the Community Land Model version 2 (CLM2). On a regional scale, the results showed that the EnKF method slightly improved the model in high-elevation locations with little violation of the mass balance. While the rule-based approach was more efficient in low elevation regions, it resulted in a significant disruption of the water balance.

In another snow cover assimilation study [39] assimilated MODIS SCF into the CLM version 4 [52,53] using an Ensemble Adjustment Kalman Filter (EAKF; a variant of the EnKF) and successfully corrected the model’s tendency for early snow melt. Moreover, [42] used MODIS SCF and the Interactive Multisensor Snow and Ice Mapping System (IMS) snow cover product (see Section 4.1.2) as constraints for the assimilation of passive microwave SWE retrievals into the Noah land surface model. More recently, [45] used MODIS snow cover albedo to improve multi-layer model estimates of snow depth and SWE at the local scale. Additionally, [54] assimilated Landsat SCF over several Andean basins and showed significant improvements (increased correlation and reduced mean error and RMSE) of SWE estimates compared to model estimates without assimilation. Finally, particle batch smoother assimilation approaches [40,41,43,46] based on a coupled land surface model and snow depletion curve model have also been used to assimilate SCF into land surface models and improve SWE estimates.

The present study focuses on snow cover assimilation into the NASA Catchment land surface model (CLSM, [55,56]). CLSM is the land surface model of the suite of widely-used reanalysis products from the Modern-Era Retrospective Analysis for Research and Applications (MERRA, [57–60]). The CLSM of the original MERRA version (hereinafter referred to as CLSM-MERRA) underwent some parameter changes to address known limitations. The updated version of CLSM is used in MERRA-Land [58] (hereinafter referred to as CLSM-MLand). A slightly modified version of CLSM-MLand is used in the currently operational MERRA-2 system [59,60]. Among the parameters that were changed is the minimum SWE in the snow-covered area (SWE_min) (see [60] for a detailed list of CLSM changes between MERRA, MERRA-Land, and MERRA-2). This parameter change, in particular, impacts SCF estimates in CLSM-MLand such that more snow is needed in CLSM-MLand before the hydrological catchment is considered to be fully snow-covered.

The objective of the present study is two-fold. First, we assess the skill of SCF estimates from CLSM-MERRA and CLSM-MLand versus MODIS observations. Second, we design a simple and computationally efficient algorithm to assimilate MODIS SCF observations into CLSM in order to improve the modeled SCF and, possibly, snow depth and SWE estimates. Since the snow depletion curve of CLSM is relatively simple and does not lend itself for use with the EnKF (or its variants), we propose a novel set of empirical rules to assimilate MODIS SCF observations into CLSM. By considering the difference (or misfit) between the observed and modeled SCF in the computation of the SWE updates, our approach does not consider the observations to be perfect. That is, in contrast to the direct insertion approaches described above [35,36], the new approach described here implicitly considers errors in the observations, as well as the model estimates. However, our approach does not rely on an ensemble, in contrast to the ensemble-based methods described above [39–41,43–46].

The paper is organized as follows: The land surface model and the MODIS SCF data are described in Section 2. This section also includes the assessment of CLSM-MERRA and CLSM-MLand versus MODIS snow cover observations to provide a baseline understanding of the model’s skill. Next, Section 3 discusses the assimilation algorithm and Section 4 is dedicated to the validation data sets and approach. The assimilation results and discussion are provided in Section 5. A summary and conclusions are presented in Section 6.

## 2. Model and Data

### 2.1. Model Description

CLSM uses hydrological catchments known as “tiles” with boundaries defined by the topography and stream topology. Energy and water balance equations are computed within each tile of the model. A three-layer snow module incorporates snow physics including densification, snowmelt, and refreeze [61]. The model uses an important parameter, SWE_min, which describes the minimum SWE that must be present per unit surface area before the model considers the surface to be snow covered. If the amount of snow on a given tile is insufficient to cover the entire tile with at least an amount of SWE_min, the SCF is the fraction of the tile area that would be covered by an amount equal to SWE_min, that is, SCF = min (1, SWE/SWE_min). When there is surface melt or when rain falls on existing snow, water can percolate into the lower snow layers, where it may refreeze. The density of each snow layer is modeled as a function of air temperature and compaction due to the over-burdening weight of over-layering snow. Each layer of newly-fallen snow is initially given a density of 150 kg m^−3^, which increases as the snow ages. Snow depth is diagnosed by dividing SWE by its density.

The prognostic state variables for each of the three snow layers include SWE, snow heat content, and snow depth. The value of SWE_min was 13 kg/m^2^ (or, equivalently, 13 mm) in CLSM-MERRA and was changed to 26 kg/m^2^ in CLSM-MLand. This larger value is still used in MERRA-2. Other parameters also changed (see Table 2 of [58] and Table 2 of [60]), including the capacity of the canopy interception reservoir, the areal fraction of canopy leaves onto which large-scale precipitation falls, the areal fraction of canopy leaves onto which convective precipitation falls, and the maximum depth of the uppermost snow layer (Zmax). The changes to SWE_min and Zmax were made to improve the modeled snow albedo and the stability of the surface energy balance calculation in snow covered areas. The doubling of the SWE_min parameter from CLSM-MERRA to CLSM-MLand has a significant impact on the SCF such that more snow is needed in CLSM-MLand before the catchment can be fully snow-covered.

In this study, both CLSM-MERRA and CLSM-MLand are used in off-line (land only) simulations forced with identical meteorological forcing fields extracted from the original MERRA data product [57]. The forcing data are available at an hourly time step and a spatial resolution of 1/2° × 2/3° in latitude and longitude, respectively. While model forcings are provided hourly, the LSM is integrated forward in time at a 20-min time step. The precipitation field used was bias-corrected using the Global Precipitation Climatology Project (GPCP) precipitation gauge and satellite-based product version 2.1 [62,63].

### 2.2. MODIS Snow Cover Fraction Observations

In this study, MODIS-derived SCF data are first used to assess the (model-only) catchment-derived SCF output and then assimilated into CLSM (with assimilation outputs validated against independent observations, see Section 4). MODIS-derived SCF data was successfully used in earlier studies to improve land surface model estimates of snow at the continental and global scales [35,36,38,39]. However, [32] showed that MODIS data can be systematically biased compared to Landsat ETM+ snow cover data in mountainous regions. The average RMSE between Landsat ETM+ and MOD10A1 snow cover fraction over the Colorado Rocky Mountains, the Upper Rio Grande, California’s Sierra Nevada, and the Nepal Himalaya was 0.23. We used SCF data from the Version 5 MOD10C1 product, which is based on binning the daily, 500-m MOD10A1 snow-covered and snow-free land observations into corresponding cells of the 0.05-degree Climate Modeling Grid (CMG) [20]. The MOD10C1 product also includes a daily confidence index (CI) field, cloud cover field, and snow spatial quality assurance percentage (QA). The CI is defined as the percentage of clear-sky land observed for the day. The snow spatial QA field provides additional information on the usefulness of the snow cover data [64]. MOD10C1 data are available via the National Snow and Ice Data Center (NSIDC) web site. In this study, model outputs were compared against MODIS observations for the period from 1 September 2001 to 1 September 2009 (eight years). MOD10C1 has been extensively validated against independent observations [24,27,65,66].

The daily 0.05° CMG MODIS data were aggregated to a 1/2° latitude by the 2/3° longitude grid coincident with the spatial resolution of MERRA forcing fields and gridded catchment model output. During this aggregation, only 0.05° CMG grid cells with a daily percentage of snow greater than zero and a CI greater than 20% were used to compute the mean snow cover fraction. Otherwise, the grid was filled with missing data. The choice of the CI threshold of 20% is larger (i.e., more conservative) compared to the value of 6% used in [35]. Daily, aggregated MODIS SCF data on the 1/2° latitude by the 2/3° longitude grid were used as observations during the assimilation process. Daily SCF data were also averaged into monthly data and later compared against monthly average CLSM-MLand to assess the impact of the parameter changes on the model (Section 2.3). The monthly aggregation was performed to minimize the effects of cloud cover while accommodating data gaps in the MODIS snow data during the evaluation of areal snow cover extent (SCE (m^2^)) based on SCF (%) estimates.

### 2.3. Evaluation of CLSM-MERRA and CLSM-MLand Snow Cover

**Figure 1.**
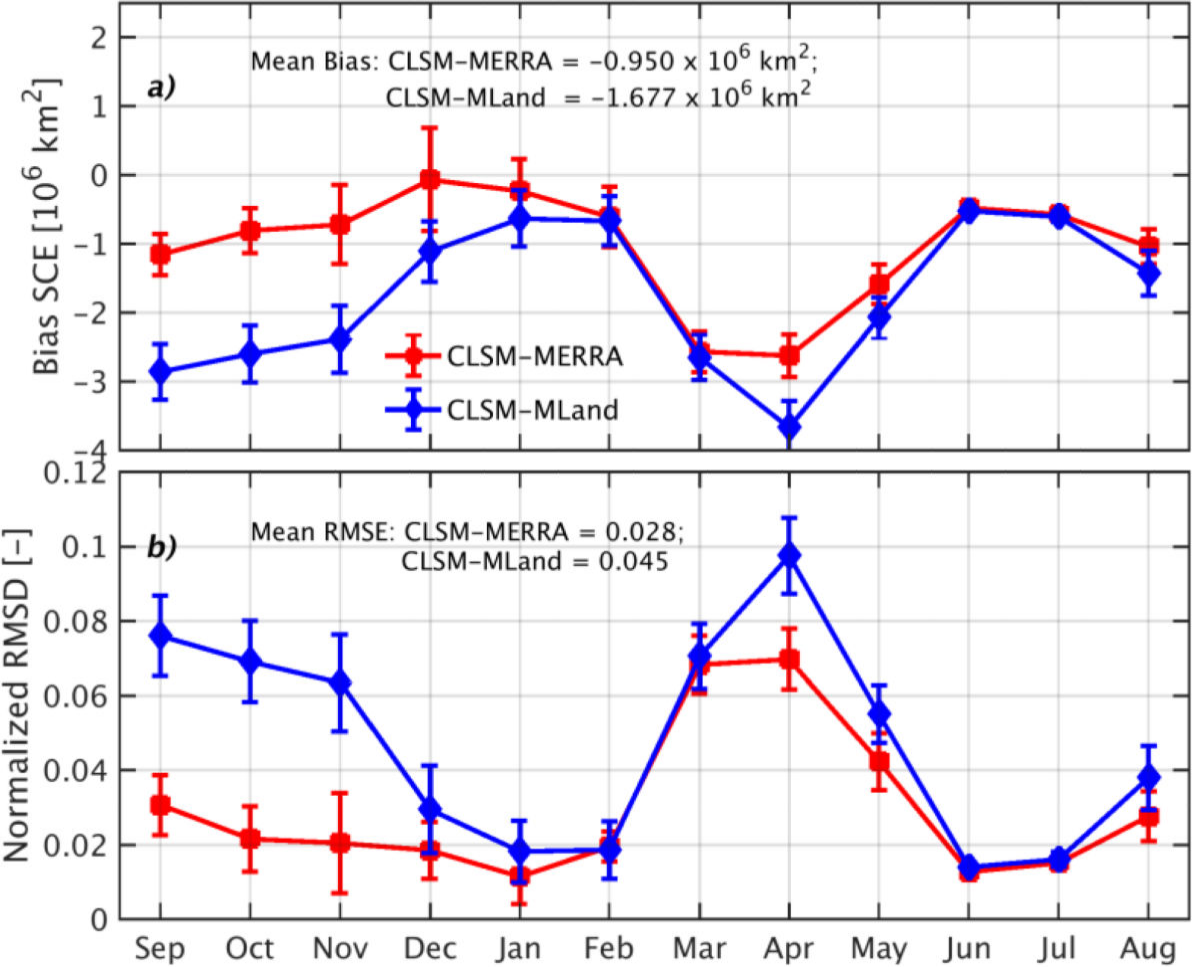
(**a**) Bias and (**b**) normalized RMSD of monthly SCE from CLSM-MERRA and CLSM-MLand for part of the Northern Hemisphere poleward of 35°N for the period 1 September 2001 to 1 September 2009. Metrics are computed vs. monthly MODIS SCE observations. Error bars represent the standard deviation across space. Normalization of the RMSD in (**b**) is by the maximum annual MODIS SCE.

To provide insights into the skill of SCF estimates from CLSM and to complement the discussion in [58,60], this section evaluates SCE estimates from CLSM-MERRA and CLSM-MLand vs. MODIS-derived SCE. Figure 1 highlights the monthly SCE (a) bias and (b) normalized root-mean-square differences (RMSD) vs. MODIS for the Northern Hemisphere poleward of 35° N during the period 1 September 2001 to 1 September 2009. RMSD was normalized by the maximum annual MODIS SCE. CLSM-MERRA showed small negative bias vs. MODIS (less than 10^6^ km^2^ in magnitude) during the accumulation phase (September–February) followed by larger, negative biases during the ablation phase from March to May (Figure 1a). The normalized RMSD remains at approximately 0.03 [−] during the accumulation phase and reaches a maximum of 0.065 [−] during ablation (Figure 1b). In the annual average, CLSM-MERRA underestimates MODIS SCE by −0.950 × 10^6^ km^2^, with a normalized RMSD of 0.028 [−].

The seasonal progression of the CLSM-MLand bias and normalized RMSD is similar to those of CLSM-MERRA but with a more negative bias and larger normalized RMSD during most months, except for January–March, when there was no significant difference between the two models. This period corresponds to the peak snow accumulation when the majority of the grid cells are 100% snow covered and the effect of the SWE_min parameter choice in the two model versions is minimized on a relative scale. The annual average CLSM-MLand bias and normalized RMSD are −1.677 × 10^6^ km^2^ and 0.045 [−], respectively, which represents a 76% and 61% deterioration relative to the CLSM-MERRA bias and normalized RMSD, respectively. The differences between the skills of CLSM-MERRA and CLSM-MLand can be largely attributed to the change in the SWE_min parameter. These findings are consistent with those of [60].

## 3. Assimilation Algorithm

**Figure 2.**
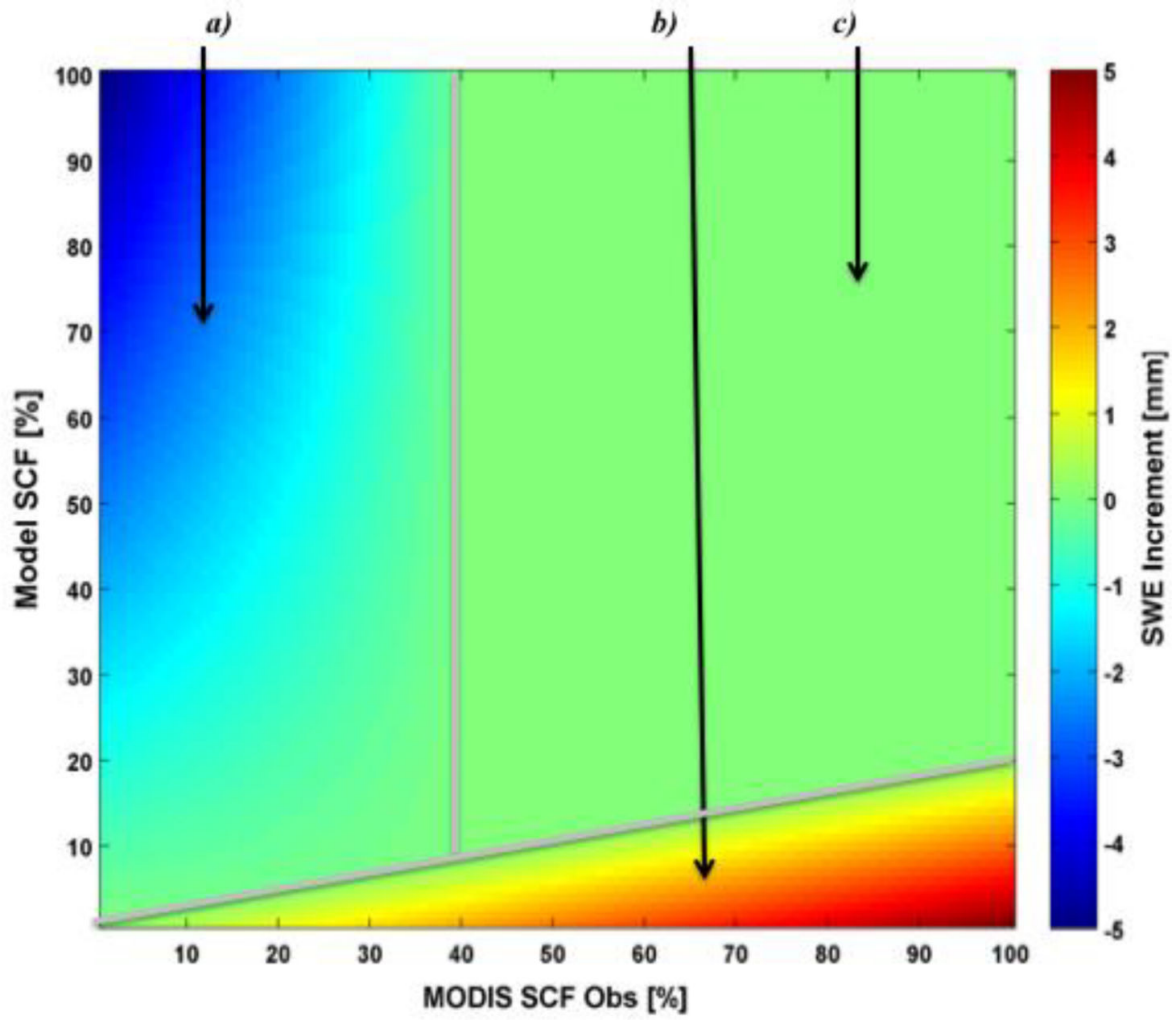
Graphical representation of the empirical formulation used to compute the SWE increment during assimilation of daily MODIS SCF for $incr_{SWE}^{Max}$ = 5 kg m^−2^, SWE_min = 26 kg m^−2^, α = 0.2 [−], and β = 40%. Blue (**a**) corresponds to snow removal, red and yellow (**b**) represent the addition of snow and, green (**c**) corresponds to when there is no significant difference between the model and the observations.

Since the snow depletion curve in CLSM is too simplistic to use as an observation operator in Kalman filter-based data assimilation, we used an approach of intermediate complexity between the simple direct insertion technique of [35] and a computationally intensive Kalman filter-based assimilation technique [48]. We, therefore, refined the rule-based approach of [35], which was based on binary differences between the observed and modeled snow cover and only considered the presence or absence of snow. Here, we define an empirical gain function that depends on the (continuous) difference between the observed and modeled SCF. This gain function, illustrated in Figure 2, determines how much snow should be added or removed in response to any discrepancies between the observed and modeled SCF. The technique does not explicitly describe model or meteorological forcing uncertainties and does not use an ensemble. Rather, the difference between the model and satellite-derived SCF is used as an indicator to guide the correction of the model estimates of SWE and, hence, SCF. That is, the calculation of the magnitude of the SWE correction (increment) implicitly takes into account errors in the simulated and the observed SCF.

An empirical set of equations was obtained based on insight in the snow model structure and by tuning some design parameters using trial and error (see also Section 5.5). The figure shows the analysis increment for the snow water equivalent (Δ*SWE*) as a function of observed SCF on the x-axis and modeled SCF on the y-axis. If the modeled snow cover fraction (*SCFm*) is less than a constant factor *α* times the observed snow cover fraction (*SCFo*) (corresponding to region b in Figure 2), an increment that is proportional to *SCFo* and inversely proportional to *SCFm* is added to the modeled SWE. Snow is removed whenever the observed SCF is below a threshold *β* (%) and *SCFm* ≥ *α SCFo* (corresponding to region a in Figure 2). In this case, the (negative) SWE increment is proportional to the modeled SWE and inversely proportional to the observed SCF. Outside of these two regions (corresponding to region c in Figure 2), the difference between *SCFm* and *SCFo* is considered small enough for the SWE increment to be zero. Formally, the empirical gain can be written as:

(1) $$\Delta SWE=\{\begin{array}{cc} incr_{SWE}^{Max}(SCF_{o}-\frac{SCF_{m}}{\alpha }), & \\ & SCF_{m}<\alpha SCF_{o}\\ -SWE_{m}(100-\frac{SCF_{o}}{\beta }), & \\ & SCF_{o}<\beta \\ 0 & \\ & \mathrm{otherwise} \end{array}$$

where *ΔSWE* is the modeled SWE increment and *α*, *β*, and $incr_{SWE}^{Max}$ are design parameters. The maximum SWE increment ($incr_{SWE}^{Max}$) is set to 5 kg m^−2^ (or (mm)) consistent with [35]. Further details concerning the choice of the maximum SWE increment are provided in Section 5.5.

An important feature of the empirical algorithm is that it can easily be adjusted for the change in the SWE_min model parameter. The threshold for MODIS observations below which snow may be removed from the model is β, and αSCF_o_ is the line below which snow may be added to the model. As discussed in Section 2.2, the modeled SCF is more consistent on average with MODIS observations in CLSM-MERRA (SWE_min = 13 kg m^−2^) compared to CLSM-MLand (SWE_min = 26 kg m^−2^). When the CLSM-MLand version is used, the modeled SCF is systematically underestimated. The difference between the CLSM-MERRA and the MODIS SCF rarely exceeds 40 %. We therefore set β = 40% and α = 0.4 [−] when CLSM-MERRA is used. To compensate for this bias (due to the change in SWE_min), we use α = 0.2 [−] when MODIS SCF observations are assimilated into CLSM-MLand.

## 4. Evaluation Datasets and Approach

### 4.1. Evaluation Datasets

The three datasets used to evaluate the MODIS SCF data assimilation results are the Canadian Meteorological Centre (CMC) Daily Snow Depth Analysis product, the IMS Snow Cover product, and the ground-based observations of SWE from the Snow Telemetry (SNOTEL) network.

#### 4.1.1. Canadian Meteorological Centre (CMC) Daily Snow Depth Analysis Data

The CMC snow data product consists of snow depth across the Northern Hemisphere [67,68] and is available from the NSIDC web site. CMC snow depth is based on a six-hourly optimal interpolation of a snow model with in situ snow depth reports from the World Meteorological Organization (WMO) information system. A simple snow model forced by precipitation and analyses of screen-level temperature fields [67] provides the initial background field. In areas where there are no snow depth observations, the reported snow depth corresponds to the modeled background estimate. The average altitude of snow stations used to condition CMC data is biased toward low-elevations (<400 m); therefore, CMC data are considered unreliable at high altitudes and likely negatively biased relative to actual snow depths. The data have been widely used to evaluate model snow outputs (see [58,60,69,70]). The CMC data have a horizontal resolution of approximately 24 km (706 × 706 pixels) projected onto a polar stereographic grid. The CMC snow depth estimates were converted to SWE estimates using the snow density parameterization of [71].

#### 4.1.2. Interactive Multisensor Snow and Ice Mapping System Snow Cover

The IMS snow cover product [72] provides estimates of daily snow and ice cover extent over the northern hemisphere. IMS data are available from NSIDC at a horizontal resolution of 24 km from February 1997 to the present. A higher resolution (i.e., 4 km) daily product is available from February 2004 to the present, but was not used in this study because pre-2004 measurements were required. Inputs to the IMS product include satellite data sources from visible (VIS) and infra-red (IR) satellite imagery including measurements from the Polar Operational Environmental Satellites (POES), Geostationary Orbiting Environmental Satellites (GOES), the Geostationary Meteorological Satellite (GMS), the European Weather Satellite (METEOSAT), and the Advanced Very High Resolution Radiometer (AVHRR). While MODIS is also used during the production of the IMS snow cover product, it is secondary relative to the platforms listed above. Band 1 (620–670 nm) was used for that purpose starting in February 2004 while bands 4 (545–565 nm) and 6 (1628–1652 nm) were used in the production of the MODIS MOD10C1 product. In this study, NOAA IMS and MODIS SCF are considered independent from one another.

Microwave satellite data from the US Department of Defense (DOD) polar orbiters, and the Defense Meteorological Satellite Program (DMSP) were also incorporated to allow an estimate of snow cover in the presence of clouds. The IMS algorithm uses a threshold-based decision-tree technique to combine all clear-sky pixels in a VIS/IR map. The map from the previous day is used to fill in any remaining undetermined pixels [73].

#### 4.1.3. Snow Telemetry (SNOTEL) Observations

We used ground-based SWE from the SNOTEL network [74] to evaluate model performance in the Western U.S. regions. Measurements of SWE by SNOTEL were acquired by the Natural Resources Conservation Service (NRCS) of the U.S. Department of Agriculture primarily in the Western United States. A total number of 702 stations were screened according to the masking and minimum data requirement as described further below. Altitudes of the stations range from 6 to 3542 m above mean sea level (a.s.l.) with an average altitude of 2122 m a.s.l. Pressure-sensing snow pillows are used to automatically measure SWE changes on an hourly basis. The stations are mostly located at high altitudes with high snow accumulation. The average SWE across all stations during the study period ranged from 0.25 to 768 mm. Although there are scale and spatial sampling issues with SNOTEL network data [75–77], the data provide invaluable ground-based SWE observations for snow analyses and model validation in the U.S., as illustrated in regional-scale observational studies [74,78–80] and validation of LSMs [77,81,82].

### 4.2. Evaluation Approach

A number of statistical metrics were computed including bias, RMSD, spatial correlation coefficient (R), and the Fisher r-to-z transformation test. The latter was used to determine the significance of the difference between two independent correlation coefficients. A summer mask (excluding June–July– August–September) and minimum data requirement (<5% of Non-Not-a-Number (NaN) values across the eight-year daily series) were applied to CMC and SNOTEL daily data prior to the computation of RMSD and R. Furthermore, we used four categorical forecast verification metrics to quantify the performance of the assimilation routine against the IMS SCF product: (1) proportion correct (PC); (2) probability of detection (POD); (3) false alarm rate (FA; probability of false detection); and (4) “binary” snow cover fraction (SCF_binary_). These metrics were calculated according to Equations (2)–(5) using the contingency table elements (a, b, c, d) defined in Table 1.

(2) $$\text{PC}=\frac{\text{a}+\text{d}}{\text{a}+\text{b}+\text{c}+\text{d}}$$

(3) $$\text{POD}=\frac{\text{a}}{\text{a}+\text{c}}$$

(4) $$\text{FA}=\frac{\text{b}}{\text{b}+\text{d}}$$

(5) $${\mathrm{SCF}}_{\text{ binary }}=\frac{a+b}{a+b+c+d}$$

Categorical statistics between the IMS snow cover product and the snow cover estimate from CLSM-MLand without data assimilation (a.k.a. open-loop, OL) and with data assimilation (DA) were calculated across the eight-year study period (excluding the summer months). For POD and PC, the higher the score, the better, with a perfect score equal to one. For FA, on the other hand, a lower score is better, with a perfect score equal to zero.

## 5. Results and Discussion

CLSM-MLand simulations serve as the OL in the following discussion. The assessment of CLSM-MLand SCF output shows that the model tends to underestimate SCF (Figure 1). The goal of the data assimilation (DA) routine used here was to improve snow detection and, in the process, presumably improve snow depth and SWE estimates. The results from the assimilation routine were evaluated using the IMS daily snow cover product, CMC daily and monthly snow depth and SWE estimates, and daily ground-based SWE (SNOTEL) observations.

### 5.1. Comparison between IMS Snow Cover Product and Assimilated Snow Cover Fraction

**Figure 3.**
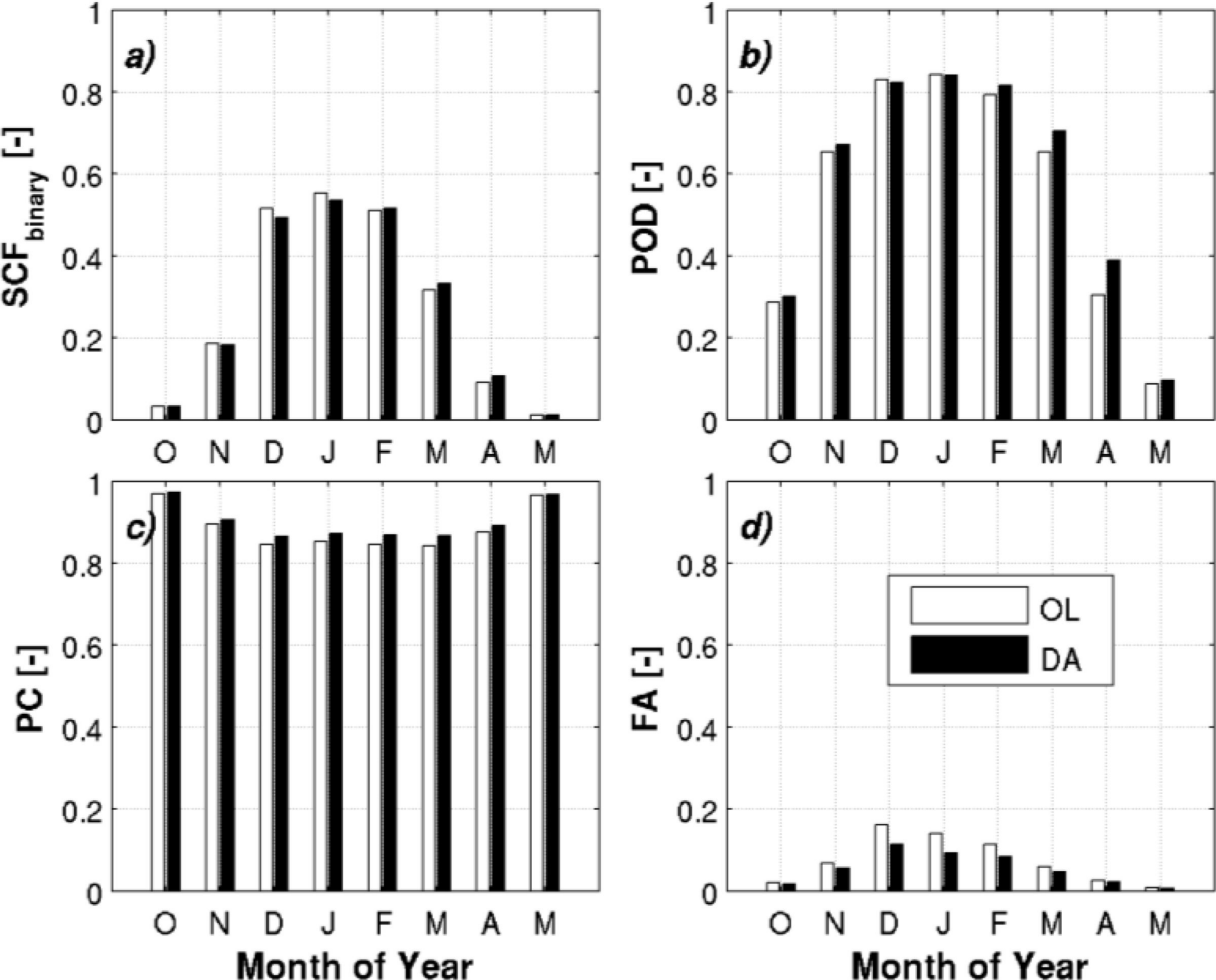
Comparison of open-loop (OL) and data assimilation (DA) estimates relative to IMS snow cover extent across the conterminous US north of 35 degrees latitude for October 2001 to October 2009 where (**a**) represents the average binary SCF (*SCF_binary_*); and (**b**) the probability of detection (POD); (**c**) proportion correct (PC); and (**d**) the false alarm rate (FA).

Figure 3a shows the “binary” total SCF for both the open-loop and the data assimilation. Results indicate that the data assimilation reduces SCF in December and January, thereby reducing the false alarm rate (Figure 3d). Furthermore, data assimilation yields improvements (except in December and January) in the probability of detection (ranging from 2.8% to 28% of the open-loop’s probability of detection) and proportion correct (ranging from 0.3% to 2.9% of the open-loop’s proportion correct) (Figure 3b,c). Moreover, a reduction of the false alarm rate ranging from 0.9% to 5.8% of the open-loop’s false alarm rate (Figure 3d) was also observed, with the greatest reductions occurring in December (5.8%), January (5.6%), and February (3.7%) when snow cover extent is at its maximum. Overall, the assimilation of MODIS SCF improved the modeled estimate of snow-covered area vs. the IMS observations.

**Figure 4.**
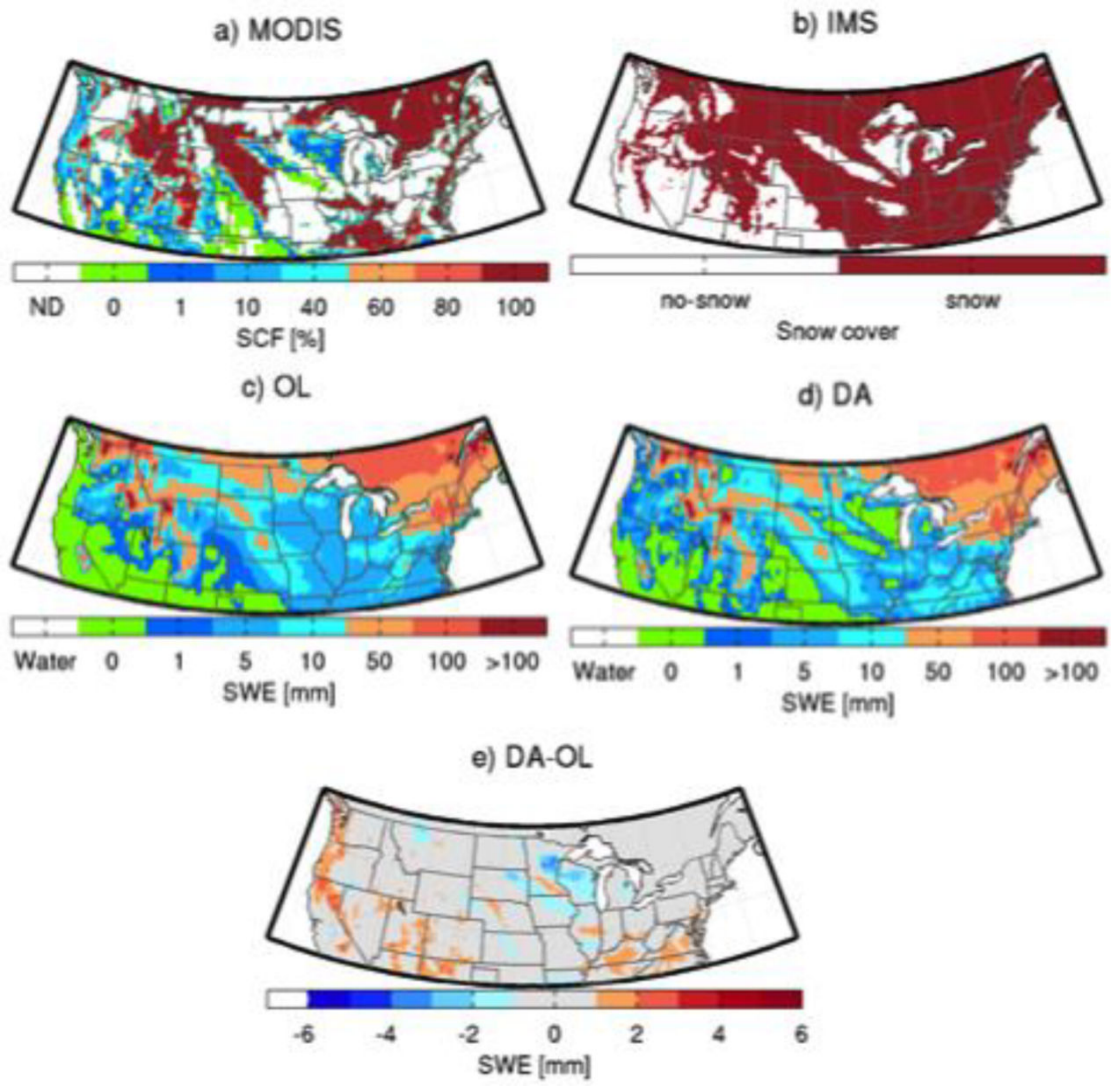
Maps of (**a**) MODIS snow cover (%); (**b**) IMS snow cover; (**c**) open-loop (OL) (model control run) SWE (mm); (**d**) model analysis (DA) SWE (mm) and; (**e**) the difference between DA and OL SWE (mm) for 17 January 2003.

**Figure 5.**
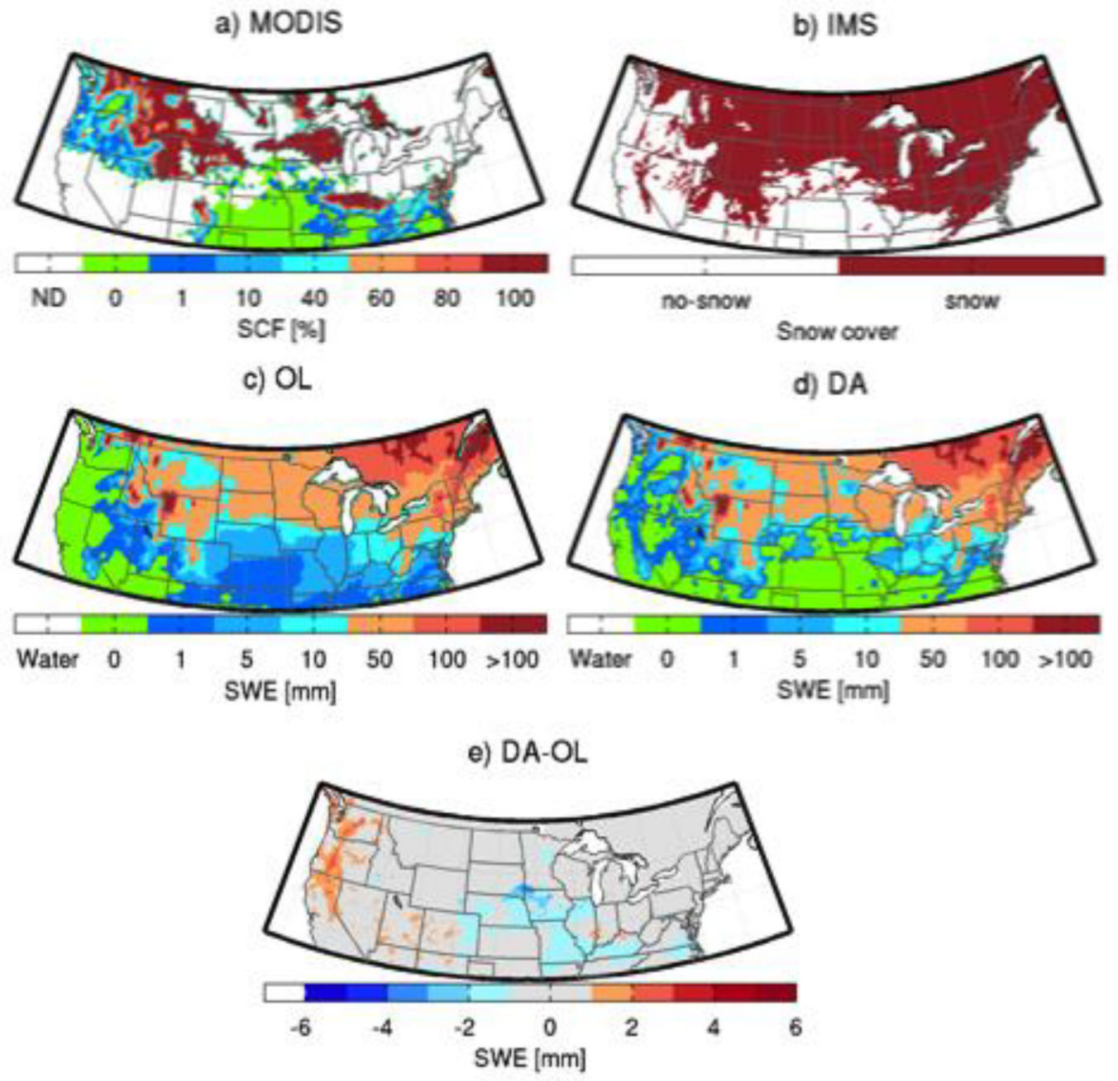
Same as in Figure 4 except for 12 February 2003.

Figures 4 and 5 help illustrate the impact of MODIS SCF assimilation on the spatial distribution of snowpack estimates on 17 January 2003 and 12 February 2003, respectively. On 17 January 2003, the MODIS observations (Figure 4a) were cloud-free in most of the western regions, Eastern US, Southeastern Canada, Kentucky, Tennessee, Ohio, Indiana, and Illinois. There is an agreement between the MODIS observations and IMS observations in those cloud-free areas (Figure 4b), except along the Pacific Coast, where MODIS shows a presence of thin snow cover (SCF < 1%). The disagreement is likely due to small differences in the threshold (relative to MODIS) used to create the IMS snow cover product. The open-loop (OL) (Figure 4c) prediction agreed well with the MODIS observations, except along the Pacific Coast, in portions of the Rocky Mountains, and the Southeastern US where the model missed the snow, and in some parts of Wisconsin and Iowa where the model had false alarms. The update (Figure 4d) successfully added snow in areas with misses and removed the superfluous snow predicted by the OL (Figure 4e). The same process occurs on 12 February 2003 (Figure 5a–e). Not only did the assimilation scheme reduce false alarms (Figure 5e), but it also added snow in areas where the model was not originally predicting snow cover (e.g., part of the Rocky Mountains). The DA results agree better with the IMS product on both days. The impact of the assimilation on SCF on those selected days is similar to that of the assimilation performed in [35].

### 5.2. Comparison between Canadian Meteorological Centre (CMC) Snow Depth and Water Equivalent (SWE) and Model Estimates

**Figure 6.**
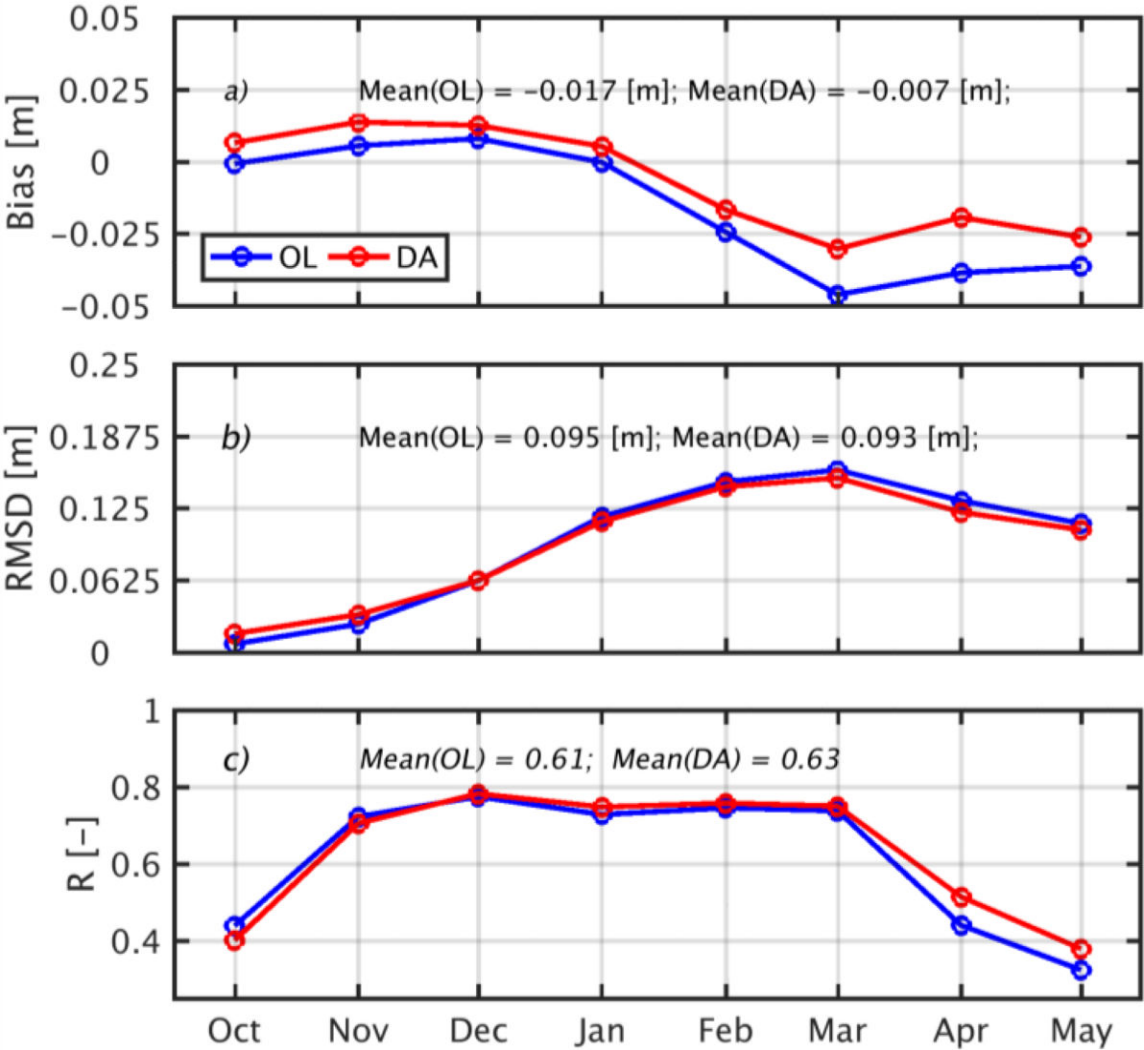
Seasonal variation of OL (blue) and DA (red) versus CMC snow depth showing: (**a**) bias; (**b**) RMSD and; (**c**) spatial correlation coefficient (R) across the conterminous US north of 35 degrees latitude for the period October 2001 to October 2009.

In this section, we assess the impact of assimilation on modeled snow depth and SWE by comparing the OL and DA snow depth and SWE against CMC-based estimates of snow depth and SWE. Figure 6 highlights the bias, RMSD and correlation vs. monthly-averaged CMC values of snow depth for both the OL and DA estimates. CMC agrees reasonably well with OL during the accumulation phase from November through January. From January to May, OL, in general, underestimated the snow depth. DA improved the snow depth estimates as demonstrated by the reduction of the bias by approximately 0.024 m in April and May. The OL had an annual average bias of −0.017 m and RMSD of 0.095 m. While DA successfully improved this bias to −0.007 m, there was no significant change in the RMSD (from 0.095 m for OL to 0.093 m for DA). The time-average of the spatial correlation coefficient is also improved slightly from 0.61 to 0.63 between the OL and the DA. The result of the Fisher’s r-to-z transformation test of the two correlation coefficients (z = −0.22 and *p* = 0.83) means the correlations are not statistically significantly different from one another at the 5% level.

The evaluation of SWE showed no significant difference between the OL and the DA SWE bias and RMSD (not shown). There was a slight improvement in the spatial correlation coefficient from 0.59 for the OL to 0.61 for the DA, but, again, the correlations were not statistically different from one another (z = −0.21 and *p* = 0.83). A possible explanation for the insignificant difference between correlations is that most improvements obtained from the DA are in the Rocky Mountains, which coincide with areas where CMC data are considered unreliable because of the scarcity of high altitude in situ data used to condition CMC data [83]. Additionally, interpolation of data with different intrinsic scales of spatial variability in mountainous regions can be a source of uncertainly in the CMC data. Processes, such as depth hoar formation, that often occur in areas such as the Prairies are not accounted for in the production of CMC data. Finally, the heterogeneous distribution of snow due to the wind-induced erosion and transport of snow which is prevalent in the mountainous areas and the Prairies [84,85] is not taken into account neither in the CMC, nor in the model. CMC was also shown to have a tendency to melt the snow earlier than the IMS snow product [86]. All of these could be reasons why a comparison between modeled and “observed” (analysis) snow depth values does not yield significant improvements.

### 5.3. Comparison between SNOTEL SWE Measurements and Model Estimates of SWE

**Figure 7.**
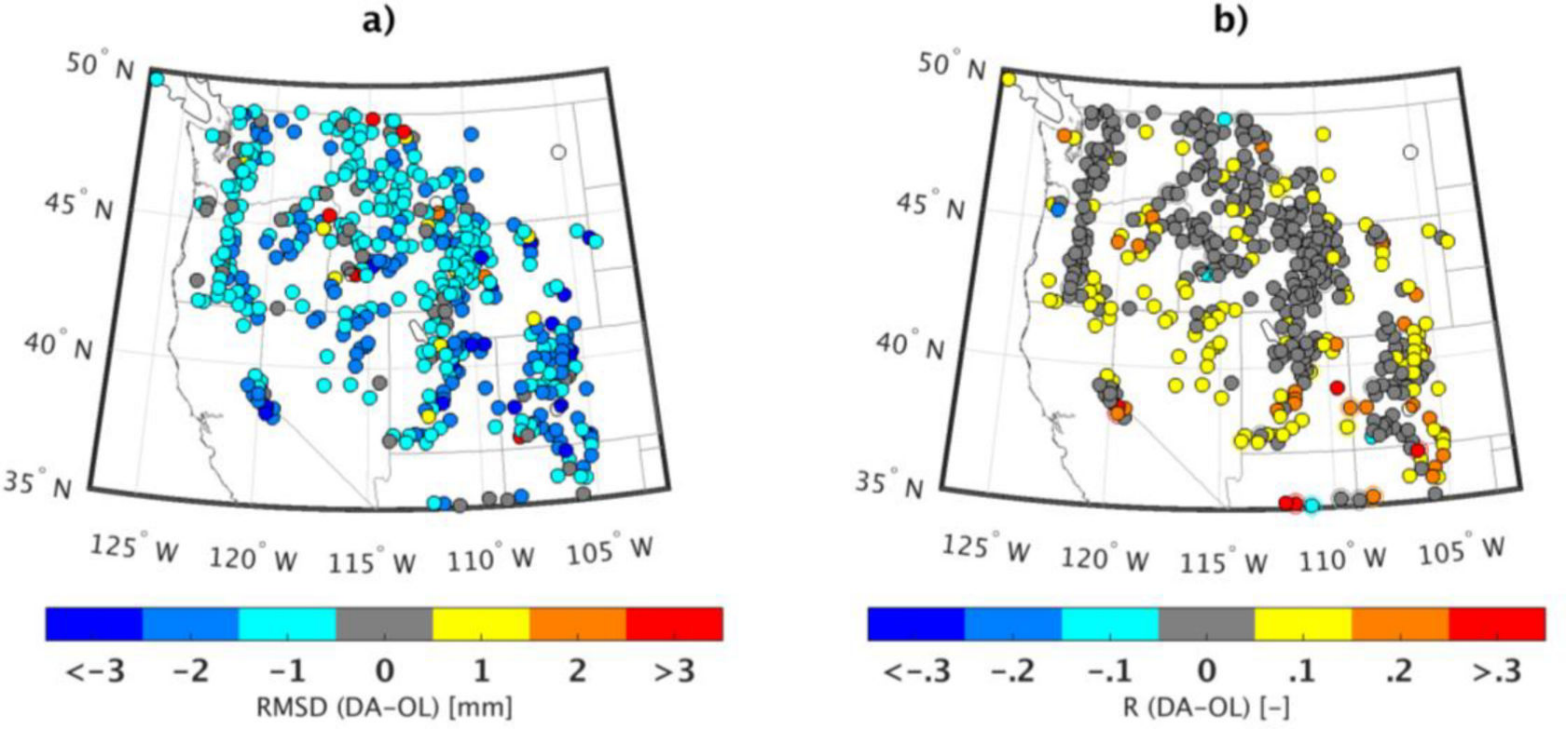
Changes in (**a**) RMSD and (**b**) correlation coefficient, R, from OL to DA (computed as DA-OL). The original DA and OL statistics were computed relative to daily SNOTEL SWE observations from 1 September 2001 to 1 September 2009.

In this section, we used ground-based observations to evaluate the performance of the assimilation routine across the Western US. An evaluation of the modeled SWE estimates against ground-based SNOTEL snow stations showed some improvements in the accuracy of SWE from the OL to DA estimates with a slight increase in correlation (0.44 to 0.49), a slight reduction in bias (−0.11 m to −0.10 m), and a slight reduction in RMSD (0.19 m to 0.18 m). The difference between DA and OL RMSD (Figure 7a) showed a small improvement (reduction) in RMSD across the majority of the study domain. A total of 95% of the stations experienced some improvement over the OL simulation. A few stations (~5% of the total stations) showed some degradation of RMSD after the DA procedure as shown in orange and red. The assimilation procedure also improved the temporal correlation (R) of the SWE compared to the SNOTEL observations (Figure 7b) for 94% of the stations. However, a few stations showed a degradation after DA (e.g., a handful of locations in Oregon, Montana, and Wyoming).

### 5.4. Assimilation Increments

**Figure 8.**
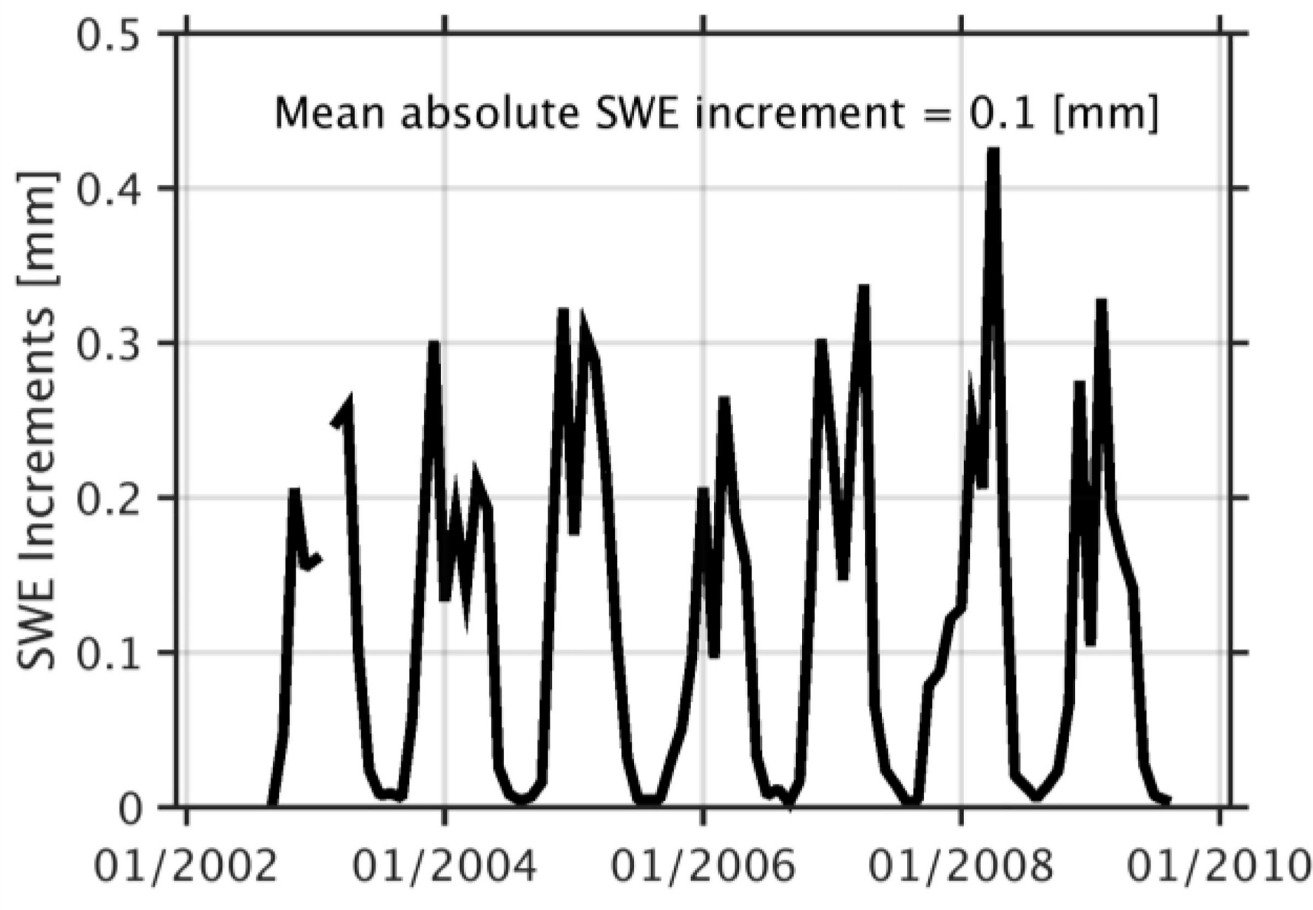
Time series of absolute values of SWE increments (i.e., the difference in DA-OL) averaged across the entire study domain, including the temporal mean of the entire simulation as shown in the title.

An investigation of the analysis increments (i.e., the difference in DA-OL) illustrates the pattern of addition and removal of snow directly attributable to the DA update. A time series of the absolute value of the SWE increments for the entire period of study averaged across the entire domain (Figure 8) highlights a seasonal cycle starting with low increments at the beginning of the snow season in September and October and steadily increasing increments during the accumulation season to reach a maximum at the beginning of the melting season. The seasonal cycle of SWE increments (not shown) indicated that snow removal tends to occur from November to March during the accumulation phase. The addition of snow via the DA update tends to occur early (September–October) and late in the snow season (April to June).

**Figure 9.**
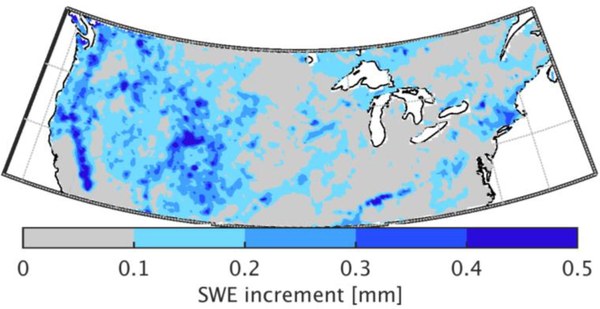
Time-average of the absolute value of SWE increments (i.e., the difference in DA-OL) across the eight-year study period.

The spatial distribution of the absolute value of the increments averaged over the study period (Figure 9) suggests that the assimilation had the greatest impact in the Western US. The effects of assimilation are marginal in the midwest, southeastern, and eastern portions of the US. The effects of assimilation in the Western US, mostly through the addition of snow, may be partly explained by negative bias in the snowfall forcings [87,88], as well as the inability of the model to adequately represent complex processes, such as sub-grid spatially-heterogeneous snowfall distribution and melting rates [89,90]. In addition, errors in precipitation estimates at high altitudes, where ground-based observations used to constrain estimates (and their errors) are sparse or non-existent, may help explain the underestimation of the snow cover in the western region [87]. The coarse spatial resolution of the model and the lack of model representation of processes, such as wind-induced snow redistribution, prevalent in the Rocky Mountains, may also explain why the DA was more impactful in those areas.

### 5.5. Sensitivity Analysis

**Figure 10.**
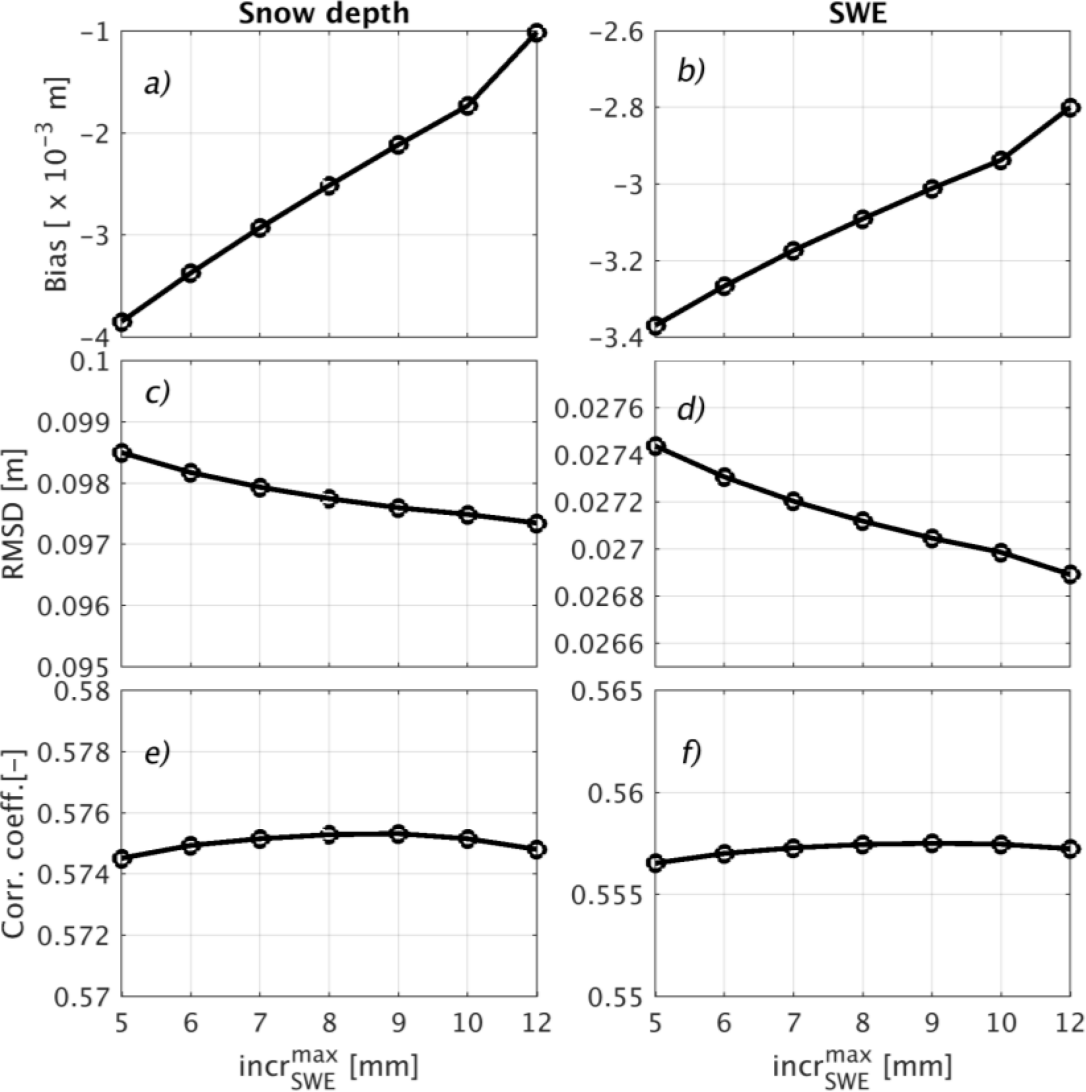
Optimization of the maximum SWE added ($incr_{SWE}^{Max}$) to a catchment. Subplots (**a**,**c**,**e**) are, respectively, the bias, RMSD, and the correlation coefficient of the DA snow depth versus CMC snow depth. Supblots (**b**,**d**,**f**) are, respectively, the bias, the RMSD, and the correlation coefficient of the DA SWE versus CMC SWE using the snow density parameterization of [71].

A sensitivity study was carried out to determine the most appropriate value of $incr_{SWE}^{Max}$ (Equation (1)) to apply during the assimilation update procedure. A set of $incr_{SWE}^{Max}$ = {5, 6, 7, 8, 9, 10, 12} mm values was tested. Figure 10 shows the RMSD and correlation coefficient of the DA results relative to CMC-based snow depth and SWE estimates. For $incr_{SWE}^{Max}$ > 5 mm, the RMSD did not significantly decrease, nor did the correlation coefficient (Figures 10) significantly increase. Moreover, in comparing the results of the different DA scenarios with the IMS snow cover product, it was found that for $incr_{SWE}^{Max}$ > 5 mm, the false alarm rate increased significantly while the POD and PC remained relatively unchanged (results not shown). Based on these results, it was concluded that $incr_{SWE}^{Max}$ = 5 mm is a reasonable approximation for the maximum amount of snow that can be added to (or removed from) a catchment during the relatively simple, rule-based update procedure.

## 6. Summary and Conclusions

In this study, we first evaluated SCF estimates from two versions of CLSM against MODIS observations. The two model versions use different values for the SWE_min parameter, which represents the minimum amount of SWE required to achieve full snow cover. The parameter values investigated here were used in the original MERRA reanalysis (CLSM-MERRA: SWE_min = 13 kg m^−2^) and in MERRA-Land (CLSM-MLand: 26 kg m^−2^), with the latter value still being used in the current MERRA-2 system. We found that snow cover estimates from CLSM-MERRA agree better with MODIS observations than CLSM-MLand. Next, we assimilated the MODIS SCF observations into CLSM-MLand using a simple and computationally-efficient rule-based data assimilation framework in which SWE increments were applied based on an empirical gain function of the modeled and observed SCF. The new approach does not require an ensemble, but does implicitly consider errors in the observations, as well as the model estimates. We assessed SCF, snow depth, and SWE output from the CLSM-MLand model with and without assimilation of the MODIS SCF observations against independent data.

The study demonstrates that the assimilation of MODIS snow cover observations improves the characterization of snow cover extent. The improved rule-based update procedure also resulted in small improvements to snow depth and SWE estimates. Specifically, the assimilation reduced the bias and RMSD of snow depth (relative to the OL) when evaluated against the CMC product. There was also a modest improvement in the spatial correlation coefficient for snow depth and SWE, although the differences in correlation between the OL and the DA are not statistically significant at the 5% level. A similar improvement in performance was obtained for most of the SNOTEL snow stations, with a slight reduction of RMSD and an increase in correlation coefficient. The update procedure had the greatest impact in the central US, which is characterized by relatively thin, and often ephemeral, snow, and in the Western US with its complex and heterogeneous topography. While previous studies (e.g., [35]) showed that the MODIS snow cover direct insertion algorithm was found to be efficient only in areas with ephemeral snow, our results demonstrate that the new algorithm can also improve snow cover estimates in mountainous regions where snow mass is a more significant contributor to the hydrologic cycle.

The current form of the empirical gain function (Figure 2) was motivated by insights into the model structure (Section 3), and our results demonstrate that, in its current form, the gain function yields improved snow estimates. However, there is no reason to believe that the gain function is optimal. Future work should address its functional form along with a refined calibration of its parameters beyond what was presented here (Section 5.5). A general drawback in the use of MODIS (or any other optical) SCF observations is their reliance on cloud-free scenes, which are infrequent in large areas of the globe, particularly in the Northern Hemisphere during winter. Another possible disadvantage is related to uncertainties in the MODIS snow cover data in dense forest cover areas. Another potential limitation of this study is the possibility of repeated cycles of adding and melting of snow in some areas with persistent warm temperature bias in the forcing data. In addition, the DA scheme had a limited impact on areas with deep and complete snow cover (Northeastern US and Southeastern Canada). In other words, MODIS SCF observations only provide limited information regarding snow mass. Other satellite observations such as passive microwave brightness temperatures could be used to improve snow depth and SWE in areas with deep snow cover through assimilation.

In a broader context, this study demonstrates the potential of using a data assimilation technique with intermediate complexity to assimilate snow cover observations into large-scale land surface models. The method introduced here falls between a simple direct insertion approach and more sophisticated and computationally-expensive methods, such as the EnKF and its variants. The new technique facilitates the assimilation of satellite-derived snow cover fraction into snow models with snow depletion curves that are too simplistic for use with ensemble-based methods. Unlike the traditional direct insertion methods, the new technique implicitly takes into account errors in the model and the observations and is a promising method for global modeling systems that use relatively simple snow models.

## Figures and Tables

**Table 1.** Contingency table of observations versus model showing the count of snow/no snow event pairs.

	MODIS Observations		
**Model**		Snow	No snow
	Snow	a	b
	No snow	c	d
